# Chemotherapy’s effects on autophagy in the treatment of Hodgkin’s lymphoma: a scoping review

**DOI:** 10.1007/s12672-024-01142-6

**Published:** 2024-07-08

**Authors:** Roro Wahyudianingsih, Ardo Sanjaya, Timothy Jonathan, Emmy Hermiyanti Pranggono, Dimyati Achmad, Bethy Suryawathy Hernowo

**Affiliations:** 1 Postgraduate Program of Biomedical Science, Faculty of Medicine, Universitas Padjadjaran, Bandung, West Java Indonesia; 2 Department of Anatomical Pathology, Faculty of Medicine, Maranatha Christian University, Bandung, West Java Indonesia; 3 Department of Anatomy, Faculty of Medicine, Maranatha Christian University, Bandung, Indonesia; 4 Undergraduate Program in Medicine, Faculty of Medicine, Maranatha Christian University, Bandung, Indonesia; 5 Department of Internal Medicine, Faculty of Medicine, Universitas Padjadjaran/Rumah Sakit Hasan Sadikin, Bandung, West Java Indonesia; 6 Department of Oncological Surgery, Faculty of Medicine, Universitas Padjadjaran/Rumah Sakit Hasan Sadikin, Bandung, West Java Indonesia; 7 Department of Anatomical Pathology, Faculty of Medicine, Universitas Padjadjaran/Rumah Sakit Hasan Sadikin, Bandung, West Java Indonesia

**Keywords:** Autophagy, Hodgkin's lymphoma, Antineoplastic Agents, DNA damage

## Abstract

**Background:**

Classical Hodgkin Lymphomas (HL) are a unique malignant growth with an excellent initial prognosis. However, 10–30% of patients will still relapse after remission. One primary cellular function that has been the focus of tumor progression is autophagy. This process can preserve cellular homeostasis under stressful conditions. Several studies have shown that autophagy may play a role in developing HL. Therefore, this review aimed to explore chemotherapy’s effect on autophagy in HL, and the effects of autophagy on HL.

**Methods:**

A scoping review in line with the published PRISMA extension for scoping reviews (PRISMA-ScR) was conducted. A literature search was conducted on the MEDLINE database and the Cochrane Central Register of Controlled Trials (CENTRAL). All results were retrieved and screened, and the resulting articles were synthesized narratively.

**Results:**

The results showed that some cancer chemotherapy also induces autophagic flux. Although the data on HL is limited, since the mechanisms of action of these drugs are similar, we can infer a similar relationship. However, this increased autophagy activity may reflect a mechanism for increasing tumor growth or a cellular compensation to inhibit its growth. Although evidence supports both views, we argued that autophagy allowed cancer cells to resist cell death, mainly due to DNA damage caused by cytotoxic drugs.

**Conclusion:**

Autophagy reflects the cell’s adaptation to survive and explains why chemotherapy generally induces autophagy functions. However, further research on autophagy inhibition is needed as it presents a viable treatment strategy, especially against drug-resistant populations that may arise from HL chemotherapy regimens.

## Introduction

Classical Hodgkin’s Lymphoma (HL) are unique among other malignant growths due to the tumors consisting of the rare malignant tumor cells / Reed-Sternberg cells surrounded by an extensive immune cell reaction. Although surrounded by immune cells, these cells create their own supportive microenvironment inhibiting apoptosis and suppressing cytotoxic killer cells [1, 2]. Previously, the origin of the Reed-Sternberg cells was unclear due to their unique immunophenotype that does not correspond to other immune cells. For example, these cells express the B-cell transcription factor PAX5 yet lack B-cell receptor expression and other B-cell markers [3, 4]. These cells were finally identified to be of B-cell origin due to the rearrangements of the immunoglobulin heavy and light chain genes [5, 6]. One of the significant pathophysiologies of the RS cells is their active expression of the NF-κB transcription factor [2]. Additionally, some cases of HL were associated with Epstein-Barr virus infection [7]. These viruses were identified to rescue crippled germinal centers B-cells destined for apoptosis. Thus contributing significantly to the early development of HL [8].

The prognosis of classical HLis excellent; up to 90% of patients at all stages can be cured [2]. Currently, treatment strategies are focused on chemotherapy combined with radiation therapy. For patients with early-stage disease, chemotherapy using ABVD (doxorubicin, bleomycin, vinblastine, dacarbazine) combined with radiation therapy is the standard practice [9]. However, radiation therapy is also associated with an increased risk of death owing to radiation-induced toxicities [2, 9]. For advanced disease HL, the most effective regimen to date is the escalated BEACOPP by the German Hodgkin Study Group, which includes bleomycin-etoposide-doxorubicin-cyclophosphamide-vincristine-procarbazine-prednisone [10, 11]. Although effective, these treatments increased the risk of secondary malignancies and substantial early morbidity [2, 9]. Therefore, novel agents such as anti-CD30 antibodies and checkpoint inhibitors are currently being researched to improve patient outcomes and reduce morbidity [2].

Despite the high cure rate, 5–10% of patients are refractory to initial treatment, and 10–30% of patients will relapse after complete remission [2, 9]. Although the exact mechanism for these relapses is unknown, one primary cellular function that has recently been the focus of attention in tumor progression is autophagy. Autophagy is an adaptive cellular process that aims to preserve cellular homeostasis under stress conditions [12, 13]. This mechanism can prolong cell life or, if exacerbated, can induce cell death. Furthermore, autophagy is one of the fundamental mechanisms associated with cancer treatment resistance [14, 15]. Several authors have reviewed the interaction between autophagy and treatment resistance of other cancer types, with some authors concluding that autophagy was able to influence the risk of developing hematological malignancies [16] and may potentially be a novel target for hematological malignancies [16, 17].

Autophagy may also play a role in the development of HL. A study examining circulating tumor DNA in classical Hodgkin Lymphoma patients found several essential proteins influencing autophagy that are mutated [18]. For example, *GNA13* and *IPTKB* are mutated in about 25% of HL patients. These two genes code proteins that directly influence the PI3K/AKT/MTOR pathway, one of the known negative regulators in autophagy function. Constitutive activation of the PI3K/AKT pathway, which generally controls the autophagy process, was detected in most classical HL cases [19, 20]. Their activation was shown to inhibit the tumor suppressor gene FOXO1 in cHL [21] and their inhibition induces cell cycle arrest and apoptosis [22]. Additionally, PD-L1 inhibitors, the novel treatment strategies of HL [2, 9, 23], were shown to promote autophagy functions in cells and may promote tumor resistance to checkpoint inhibitors [24]. Therefore, we want to answer two specific questions in this scoping review. Does chemotherapy affect autophagy function in HL, and what are the effects of autophagy on HL?

## Methods

We conducted a scoping review according to the PRISMA-ScR extension [25]. A search was conducted on the MEDLINE database using PubMed and the Cochrane Central Register of Controlled Trials (CENTRAL) to answer our primary research question. We conducted our search in November 2023 using the term (("autophagies"[All Fields] OR "autophagy"[MeSH Terms] OR "autophagy"[All Fields] OR "autophagy s"[All Fields]) AND ("hodgkin disease"[MeSH Terms] OR ("hodgkin"[All Fields] AND "disease"[All Fields]) OR "hodgkin disease"[All Fields] OR ("hodgkin"[All Fields] AND "lymphoma"[All Fields]) OR "hodgkin lymphoma"[All Fields])) OR ("autophagy"[MeSH Terms] AND "hodgkin disease"[MeSH Terms]). These search criteria were used to maximize studies reporting autophagy and Hodgkin’s disease. Two independent reviewers then screened studies before further inclusion in the full-text review. A third independent reviewer will resolve decision conflicts. The eligibility criteria for this review are shown in Table 1

**Table 1:** Eligibility Criteria used in this review

Inclusion Criteria	
Population	ANY studies, including animal studies, in vitro studies utilizing cell lines, and clinical trials
Concept	ANY intervention is acceptable so long as measures reporting autophagy function are reported
Context	Studies must be conducted on Hodgkin Lymphoma animal models, patients, or cell lines
Types of Evidence	Peer-reviewed articles. Systematic, scoping, and narrative review articles will not be included but will be searched for relevant references. Opinion articles will be excluded
Exclusion Criteria	
Articles not in English	
Full Text not available	

**Fig. 1:**
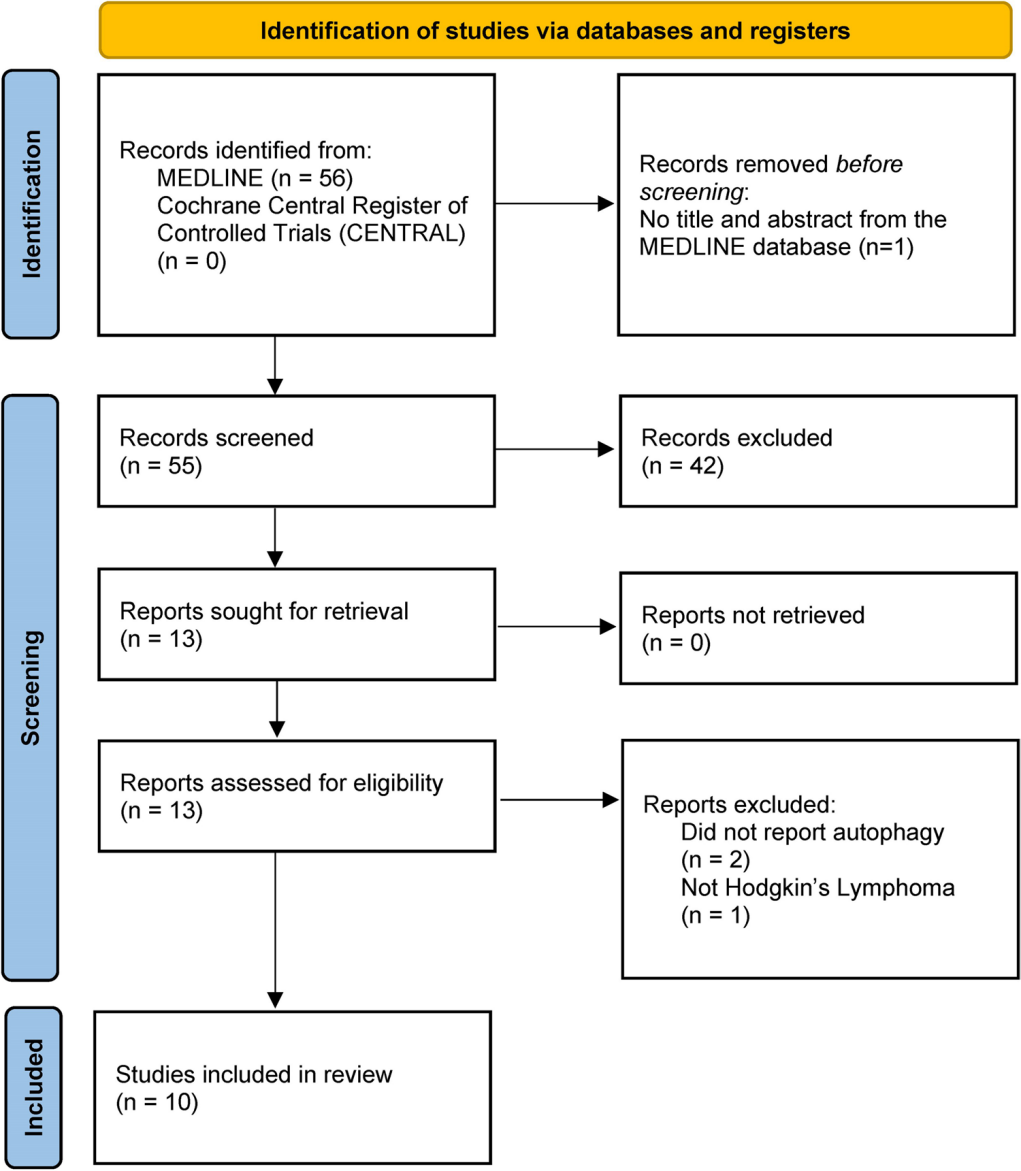
Prisma Flow Diagram. After the initial search, 56 articles were found. However, one article from the MEDLINE database has no title and abstract and, therefore, cannot proceed to the screening phase. 42 articles were excluded from the initial title and abstract screening. The full text of 13 articles was sourced and further screened. Only 10 studies were included in the review

The workflow for this scoping review can be seen on the PRISMA flow diagram in Fig. 1. The following data were extracted for articles included in the final review: article title, author names, year published, study design, intervention, and results.

## Results

Our search terms resulted in 56 articles. These articles were screened using the title and abstract, resulting in 13 articles. Following the full-text screening, 10 studies were deemed eligible, with three excluded. The reason for exclusion can be seen in the Prisma Flow diagram in the figure. The studies included were heterogeneous in their study type and results presentation and were synthesized narratively. The full data extraction of the included study can be seen in the supplementary materials. A short table showing the study characteristics can be found in Table 2.

**Table 2:** Characteristics of Studies Included in the Review

Authors	Year published	Study design	Intervention
Kyriazopoulou et al. [26]	2022	Observational, human tissue sections	None
Lin et al. [27]	2021	Experimental In vitro (L428, KM-H2) In vivo (xenograft murine models)	LMP1 transfection Doxorubicin Chloroquine
Pierdominici et al. [28]	2017	Experimental In vitro (L-428, KM-H2, L-540 and HDLM-2) In vivo (non-obese diabetic/severe combined immunodeficient mice)	DPN (ERβ selective agonist) 3-methyladenine (early autophagosome inhibitor)
Casagrande et al. [29]	2023	Experimental In vitro (doxorubicin-resistant cell lines developed from KM-H2 and HDLM-2)	Doxorubicin Chloroquine GW4869
Birkenmeier et al. [30]	2016	Experimental In Vitro (L428, L1236, KMH2, L540, and HDLM2) In Vivo (non-obese diabetic/severe combined immunodeficient mice) Observational In vivo (human lymph node samples from HL patients)	ATG5 knockdown Chloroquine
Jeong et al. [31]	2018	Experimental In vitro (L-540 and HDLM-2)	time-averaged simulated microgravity (taSMG)
Muqbil et al. [32]	2019	Experimental In vitro (KM-H2, L1236, L-428, and HDLM) In vivo (severe combined immunodeficient mice)	Ibrutinib
Yan et al. [33]	2020	Experimental In vitro (L428)	Melatonin 3-methyladenine Chloroquine
Oehadian et al. [34]	2007	Experimental In vitro (HD-My-Z)	Adriamycin Paclitaxel Bleomycin Gemcitabine
Klein et al. [35]	2013	Experimental In vitro (L428, L540 and KM-H2)	Panobinostat

All of the research included was based on pre-clinical studies exploring cell lines. Although several research studies have associated autophagy with many different forms of cancers, there is still very little evidence linking the relationship between autophagy and its effect during the treatment of HL compared to other forms of lymphoproliferative disorders. Studies that have explored autophagy in HL have yielded conflicting results. Several authors concluded that autophagy function is essential for the induction of apoptotic cell death, while others have found an unclear relationship.

Regarding the effects of chemotherapy treatment on autophagy functions in HL, one study, in particular, answered the question by screening several anti-cancer drugs on an HL cell line (HD-My-Z cells). They found that most cytotoxic drugs tested (Adriamycin, Bleomycin, and Paclitaxel) did not influence the autophagic flux on the HL cell line except for gemcitabine [34]. They found that gemcitabine administration increased the autophagic flux in HL cells. Other studies on HL cell lines also concluded that Ibrutinib [32] and Panobinostat [35], used in some cancers, induce cell death and autophagic flux. However, these data were based on preclinical studies and might not be directly translatable to clinical practice.

Although previous research showed that chemotherapy induces autophagy, the effects of autophagy on cancer cells are less conclusive. A study by Birkenmeier et al. on multiple HL cell lines identified that several critical proteins involved in autophagy, such as Beclin, lysosomal associated membrane protein 1 (Lamp1), and UNC-51-like autophagy activating kinase 1 (ULK1), are upregulated compared to non-malignant germinal center B-cell lines [30]. Indicating increased autophagy activity in HL cells. A finding confirmed by Kyriazopoulou et al. through tissue samples from patients with HL [26]. They also found that the markers for autophagy showed a positive correlation with disease relapse. However, it is unknown whether this increased autophagy activity is a mechanism for increasing tumor growth or a cellular compensation to inhibit its growth. Several studies have tried to answer this question and have yielded evidence supporting both views.

Several studies supported the notion that autophagy is essential for tumor growth. A study exploring Epstein-Barr virus infection, notorious for developing HL, found that Epstein-Barr virus (EBV) infection induces autophagy function and was robust enough to rescue cells treated with Doxorubicin. Doxorubicin is one of the current treatment regimens in HL, and their findings indicate that autophagy functions as a cellular mechanism to rescue tumor cells from apoptosis [27]. Confirming previous findings, co-administration of autophagy inhibitors was shown to reverse doxorubicin resistance in doxorubicin-resistant HL cells [29]. An extensive study on multiple HL cell lines found that inhibiting autophagy causes an increase in cell death compared to non-malignant B-cells [30]. Another study exploring the effects of melatonin administration found that this treatment induces HL cell apoptosis and autophagic flux, a finding shared by the previously mentioned study. However, interestingly, the author also tried co-treatment with autophagy inhibitors, such as 2-methyladenine and chloroquine, and found that the combination treatment significantly increases apoptosis in HL cells [33]. Taken together, these findings supported the notion that perhaps autophagy allowed cancer cells to resist cell death, mainly due to DNA damage caused by cytotoxic drugs.

Supporting the notion that autophagy is essential for cell death are several studies conducted using cytotoxic / anti-cancer drugs. Klein et al. found that using Panobinostat, a histone deacetylase inhibitor, in HL cell lines decreased cell viability dose-dependently, coinciding with increased autophagic flux [35]. Supporting their finding, another study exploring the selective activation of Estrogen Receptor Beta found that its activation reduces tumor growth and induces autophagy through overexpression of DNA Damage Regulated Autophagy Modulator 2 (DRAM2) and Microtubule Associated Protein 1 Light Chain 3 (LC3) [28]. Ibrutinib, a Bruton’s Tyrosine Kinase inhibitor, was also shown to induce cell death and increase autophagy markers such as LC3B and Autophagy Related 12 (ATG12) in HL cell lines [32]. Another fascinating study explored unconventional means of treatment for HL, simulated microgravity. They found that simulated microgravity inhibits the proliferation of HL cells [36] and was presumably caused by mitochondrial dysfunction and ROS generation, which in turn induces autophagy function [31]. These authors concluded that autophagy is an essential function in inducing cell death and an important mechanism for tumor growth inhibition.

## Discussion

The results of our scoping review can be summarized into two main topics, answering our previously stated research question. From the available evidence, we can conclude that autophagy played an essential role in HL proliferation and was induced by chemotherapeutic drugs.

A study by Oehadian et al. exploring several anti-cancer drugs on HL cell lines found that gemcitabine, but not Adriamycin, bleomycin, and paclitaxel, influenced the autophagic flux [34]. However, several problems are apparent in this study. The cell line used in the study was HD-My-Z cells, which were shown to be a misclassified cell line [37]. Additionally, their assessment and definition of autophagy are unclear and presumably subjective. Thus, we cannot reliably draw any conclusions from their study. Ibrutinib [32] and Panobinostat [35], used in some cancers, were also shown to induce cell death and autophagic flux. Although the evidence is sparse, we can conclude that the administration of chemotherapeutic drugs induces autophagy.

The induction of autophagy due to chemotherapy is probably best explained by the role of autophagy in the DNA damage response. Most conventional chemotherapeutic drugs act as anti-tumor agents by introducing DNA damage in rapidly replicating cells [38]. Examples of such drugs are Doxorubicin and Etoposide, Bleomycin, Procarbazine and Dacarbazine, and Cyclophosphamide. These drugs induce DNA damage and, in turn, reduce the tumor cell population by apoptosis. However, these cells might undergo cellular senescence or autophagy instead of cell death. Autophagy, previously known as a cellular homeostasis mechanism, preserves cellular function through energy conservation after activating the DNA damage response [39, 40]. This might explain the mechanism by which autophagy mediates treatment resistance in cancers.

Research has shown that DNA damage, either through chemotherapeutic drugs or UV damage, increases autophagy protein expression [41]. However, DNA damage can induce both the canonical and the alternative pathways in autophagy [42]. This contrasts with cellular starvation, which preferentially stimulates the canonical pathway. This increase in autophagy function due to DNA damage is mediated by several proteins involved in the autophagy and the DNA damage repair process. These authors have found that the DNA damage process induces autophagy through the inhibition of Mechanistic Target Of Rapamycin Kinase Complex 1 (mTORC1) through the ATR/Chk1 signaling [43], phosphorylation of Ulk1 [44], activation of Endonuclease G and, therefore, inhibition of mTOR [45], and through direct interaction between KU70, a protein involved in non-homologous end joining (NHEJ) mechanism of DNA repair, and ATG5 [46].

The effects of autophagy on cancer cells are, however, complex. The results from our review showed studies supporting both sides of the argument. Autophagy was shown to have protective effects on cancer cells [27, 29, 30, 33] but was also essential for cell death [28, 31, 32, 36]. The conflicting conclusion reflects autophagy's complex cellular function. On HL, the data supported the upregulation of Beclin, Lamp1, p62, and ULK1 in HL cell lines [26, 30] presumably essential for tumorigenesis, although the mechanisms are unknown. However, no research has specifically discussed their role in HL. ULK1 is a cytoplasmic kinase essential in autophagosome formation and the autophagy process [47]. Beclin is a protein involved in allosteric modulation and is essential for autophagic vesicle enucleation and autophagolysosome maturation [48]. Several studies focusing on ULK1 have found its dual role in suppressing or promoting tumor growth [47]. while Beclin has been shown to have tumor suppressor roles [48, 49]. Inhibition of ULK1 has a therapeutic potential as long as its function is properly characterized in specific cancers. A study by Egan et al. has found small molecule inhibitors of ULK1 capable of causing tumor cell cytotoxicity [50]. p62 is a selective autophagy substrate that has been directly implicated in tumorigenesis of endometrial cancer [51]. In HL, high nuclear p62 is a marker for treatment recurrences and may be a viable biomarker and treatment target [26]. However, as p62 is an autophagy substrate, treatment with autophagy inhibition may, in turn, increase p62. With its direct role in tumorigenesis, it is unclear what the effects inhibiting autophagy on p62 and treatment recurrences in HL. These findings reflected the dual role of autophagy in cancers and support its role as a potential therapeutic target that necessitates further research.

Autophagy is a fundamental cellular function that recycles harmful or unneeded cellular components or organelles [12, 52]. Two autophagy pathways were characterized due to their molecular mechanisms, the canonical and alternative pathways. These pathways function similarly, but their specific component differs; although Ulk1 is essential for both pathways, phosphorylation of Ulk1 Ser746 is crucial for alternative autophagy [44]. Additionally, the canonical pathway derived its membrane from the endoplasmic reticulum, while the alternative pathway derived its membrane from the trans-Golgi membrane [53]. p62 is a substrate of the canonical pathway only; therefore assessment of p62 cannot be used to measure the alternative autophagy pathway activity [53]. Autophagy degrades these cellular components using lysosomes, making basic molecular building blocks ready for further cellular metabolism. Autophagy formation starts in the Endoplasmic Reticulum with the creation of the autophagosome and ends during the fusion of the autophagosome and the lysosome [52]. This process is initiated by the ATG proteins, which are conserved across different species, such as yeast and mammals. Initially, the main known triggers of autophagy are cellular starvation and stress. [54] Numerous research studies have shown that inducing cellular starvation caused a change in the ATG proteins, increasing the autophagic flux [55, 56]. During these cellular crises, the function of autophagy is crucial to cellular survival by recycling damaged organelles and conserving energy during starvation. However, another less-known autophagy trigger is the DNA damage response [57, 58]. The DNA damage response is a cellular mechanism aiming to preserve genomic integrity through DNA repair, cell cycle arrest, or evoking senescence and apoptosis [39, 59]. The most important protein involved in DNA Damage is the p53 protein, which is mutated in 50% of human cancers [39, 60]. This protein was shown to interact with the autophagy process through the alternative pathway, which differs from the previously known pathway [41, 42, 61, 62].

We’ve discussed previously the difference between the alternative and canonical pathways in autophagy. However, they also differ concerning the triggers involved. One of the most researched triggers in this alternative pathway is genotoxic stress [62, 63]. Additionally, the alternative pathway does not require several essential proteins for the canonical pathway. For example, the Atg5-Atg12 proteins are unnecessary for alternative autophagy. Additionally, LC3 conversion does not happen in this pathway. Therefore, the LC3-I and LC3-II proteins cannot be used to measure autophagic flux [42, 64]. However, Ulk1 and the PI3K complexes' activity is still essential in this pathway, although with different phosphorylation locations. The activation of this pathway by DNA damage may serve several functions. The recycling ability of autophagy may be necessary to provide substrates for efficient DNA replication and repair [60]. Autophagy is also essential in regulating the cell cycle, although its interactions are extremely complex. The readers are referred to the works by Mathiassen et al. for a thorough explanation [65]. Additionally, any defects in autophagy function promote the use of error-prone DNA repair mechanisms [66].

Studies exploring the mutation of autophagy proteins supported the protective effects of autophagy on tumor cells. They found that such mutations increase cellular toxicity and death due to DNA damage caused by UV lights [38, 45, 46] and increase tumorigenesis due to DNA damage [67]. Although typically a deleterious effect, this additional toxicity may increase the efficacy of chemotherapy drugs that induce DNA damage during cancer treatment. Confirming this hypothesis, autophagy inhibition was shown to increase the efficiency of cancer therapy using DNA damage-inducing agents [68]. Several studies using doxorubicin [69], etoposide [70], and cyclophosphamide [71] showed that the inhibition of autophagy increases tumor cell death compared to controls, although none of these research explored their application in HL. These findings showed that inhibiting autophagy might be an exciting adjunct therapy for cancer treatment. However, inhibiting autophagy for all cells might prove deleterious. Proving its essential role in the DNA damage response, mutations in autophagy were shown to increase tumor susceptibility. Several of the proteins involved in the autophagy pathway were shown to be tumor suppressor proteins [72–74], essential in maintaining normal cellular growth. Several authors have reviewed the proteins involved [75–78], and the reader is referred to their works. Additionally, considering that autophagy suppression increased DNA damage [40, 45, 46, 70, 79], we can assume that autophagy suppression increased tumorigenesis by accumulating DNA damage on cells.

This duality of autophagy function in cancer prevention and formation can be illustrated by the findings derived from the study using mTOR inhibitors in cancers. The administration of rapamycin or other mTOR inhibitors was shown to delay cancer formation in cancer-prone mice infected by the Human Papilloma Virus (HPV) [80] or carrying mutations in Her2/Neu [81, 82], p53 [83, 84], PTEN [85], and other types of mutations [86, 87]. Additionally, evidence on kidney transplant patients found that using mTOR inhibitors protects de novo cancer formation, preventing up to half of the expected incidence of cancers [88–91]. Although these studies did not attribute their findings to autophagy functions, we cannot disregard the effects of mTOR inhibitions on autophagy. The increase in autophagy signaling is a potential contributor to the effects of mTOR inhibition on cancer prevention. Autophagy was shown by many studies to be involved in mediating the DNA damage response and is integral in the tumor suppression mechanism of cells [92–94].

Previous authors have reviewed that some cancer cells were addicted to autophagy in maintaining their survival [92]. This is not surprising considering the relatively hostile environment some cancer cells live in (low nutrients, low oxygen, etc.). Several studies have found that inhibiting autophagy caused an increase in radiosensitivity [95] and chemosensitivity to conventional cancer chemotherapy drugs. These studies have been replicated several times with different cell lines and cancers such as sarcoma [95], glioblastoma [96], ovarian cancer [97, 98], melanoma [99], cervical cancer [100, 101], osteosarcoma [102], colorectal cancer [103], breast cancer [100], and lung cancer [104]. One of the most extensive research by Liu et al. [105] explored mTOR inhibition on a panel of 29 human cell lines and found that the cells treated with both chemotherapy and mTOR inhibition produced a drug-tolerant subpopulation resistant to chemotherapy. However, activating the mTOR pathway eradicated these persistent cancer cell clones. Further exploration found that inhibiting autophagy selectively destroyed these persistent clones without affecting other cells. None of these studies directly examined the effects of autophagy on HL. However, from the results of our scoping review, we can derive with some confidence that since the autophagy proteins were also highly expressed in HL cells, correlated with treatment recurrence, and influenced chemosensitivity, the function of autophagy in HL is similar [27, 29, 30, 33]. These previous findings supported the notion of autophagy as one key player in the DNA damage response [53, 106]. The inhibition of autophagy increased chemosensitivity against drugs that caused DNA damage such as the chemotherapy used for HL. However, the administration of autophagy inhibitors needs to be evaluated due to their essential role in normal cells. Additionally, if the DNA damage response is the primary target we are looking for with autophagy inhibition, the risk of secondary cancers must be considered.

Several researchers, however, assume a different interpretation. These researchers concluded that autophagy is an essential function for tumor cell apoptosis. In HL, researchers have found that Panobinostat, [35] Estrogen Receptor Beta, [28] Ibrutinib, [32] and simulated microgravity [31, 36] exerted their cytotoxic effects through the induction of autophagy function. Interestingly, nearly all of these studies found that the initial administration of chemotherapy drugs increased the autophagic flux. However, none of the previous studies in HL cells explored whether inhibiting autophagy would cause a decrease in apoptosis, therefore supporting the notion that autophagy is essential for cancer cell death. We argued that perhaps the reverse is true: autophagy is necessary for cancer cell survival, and increased autophagy after treatment with such drugs is a response to the cellular stresses caused by it. Nevertheless, other studies in cancers other than HL also reported that autophagy promotion increased chemosensitivity [107] and delayed spontaneous metastasis [108], signifying the complex function of autophagy in cancer cells. Additionally, different approaches are used to inhibit autophagy in this research, such as gene knockouts [97, 98, 100, 101], small molecule inhibitors [96, 102, 104], chloroquine [96, 102], and autophagy inhibition at early stage vs. late stage [102, 109], further adding to the complexity of autophagy in cancer.

Aside from cancer cells, the tumor microenvironment also affects disease progression [110]. Therefore, autophagy function in those cells will also affect the cancer cells [111, 112]. Autophagy function in the tumor microenvironment has been shown to support cancer growth through nutrient supply and metabolic crosstalk. A study focusing on colorectal cancer cells found that autophagy expressed in cancer-associated fibroblasts enhances the proliferation of cancer cells when co-cultured together [113]. This finding is interesting since 90% of the tumor mass of HL consists of the tumor microenvironment [114]. Research has also shown differences between the tumor microenvironment of treatment responders and nonresponders in HL, although it focuses on the immunophenotypes rather than autophagy [115]. Currently, no studies have focused on exploring autophagy in the tumor microenvironment of HL tumors, which may warrant further research.

The intricacies of targeting autophagy in cancers are apparent with its inhibitory and stimulating effects on cancers. However, the current evidence on HL is lacking, with an unclear role autophagy has in HL. Research by Birkenmeier et al. has shown that HL cells overexpress ULK1 [30]. Therefore, Ulk1 inhibitors, which inhibit the autophagy process, may be a viable therapy adjunct in HL [50]. The inhibition of autophagy may increase cell cytotoxicity, especially towards DNA-damaging drugs, due to the role of autophagy in the DNA damage response. However, HL cells were also shown to have high mTOR activity. Treatment with rapamycin, a mTOR inhibitor, was shown to have cytotoxic effects on HL cell lines [116]. Although the author mentioned no effects on autophagy, the increased autophagy may affect cell apoptosis [117]. However, their findings contrast the study by Liu et al., which found the opposite: treatment with an mTOR inhibitor caused the emergence of a drug-tolerant subpopulation in cancer cell lines [105]. Therefore, the dual role of autophagy in HL may require further research characterizing autophagy’s effects in HL before autophagy inhibition becomes a viable treatment.

Based on currently available evidence, autophagy is shown to be a potent cellular adaptation mechanism influenced by cellular stresses. However, it is unknown whether they contribute to cancer survival or death. We argue that perhaps the increase in autophagy function reflects the cells' adaptation to stress caused by the drug treatment instead of an essential mechanism to cancer cell apoptosis. Yet autophagy's complex function and pathways necessitate further research identifying specific targets and their effects on cancer metastasis and secondary cancer formation. The main drawbacks of this scoping review are the small number of databases and the unavailability of grey literature due to our infrastructure not supporting access to other databases. Therefore, we might miss some evidence that supports other hypotheses. However, our results should still identify critical findings on the relationship between chemotherapy and autophagy in HL and the research gap for further exploration.

## Conclusion

Autophagy is essential in the DNA damage response and contributes to cancer cell survival. Several research studies have found the dual role of autophagy in preventing cancer cell formation and promoting cancer cell survival. The results from our review have shown that chemotherapeutic drugs were shown to induce autophagy function, presumably due to the DNA damage / cellular stress caused by such drugs. However, the effect of this increase is uncertain, with evidence supporting both views. Nevertheless, based on the evidence, we conclude that the induction in autophagy is a cell survival mechanism from tumor cells. Further research in HL is needed to ascertain its benefits, preferably through combination therapy between autophagy inhibitors and chemotherapy. Additionally, since the DNA damage response preferably activates the alternative pathway, markers measuring this pathway should be studied instead of the conventional/canonical autophagy markers. Further research on autophagy inhibition presents a viable treatment strategy, especially against drug-resistant populations that may arise from HL chemotherapy regimens.

## Data Availability

Not applicable.
